# Temporal and spatial changes in cerebral blood flow in neuropsychiatric systemic lupus erythematosus: a subtraction brain spect study

**DOI:** 10.1186/s41824-021-00112-3

**Published:** 2021-11-04

**Authors:** Ana Carolina Trevisan, Leonardo Alexandre-Santos, Rodrigo Luppino Assad, Emerson Nobuyuki Itikawa, Felipe Arriva Pitella, Mery Kato, José Henrique Silvah, Antonio Carlos Santos, Paulo Louzada-Junior, Lauro Wichert-Ana

**Affiliations:** 1 Nuclear Medicine and PET/CT Laboratory. Ribeirão Preto Medical School, Post Graduate Program in Internal Medicine, University of São Paulo, Ribeirão Preto, Brazil; 2 Magnetic Resonance Laboratory, Department of Medical Imaging, Hematology, and Clinical Oncology Ribeirão Preto Medical School, University of São Paulo, Ribeirão Preto, Brazil; 3 Division of Rheumatology, Department of Internal Medicine, Ribeirão Preto Medical School, University of São Paulo, Ribeirão Preto, Brazil; 4 Physics Institute, Federal University of Goiás, Goiânia, Goiás Brazil; 5 Inter-units Bioengineering Postgraduate Program, University of São Paulo, São Carlos School of Engineering / USP, São Carlos, SP Brazil

**Keywords:** Systemic lupus erythematosus, Neuropsychiatric form, Brain SPECT, SISCOM

## Abstract

This study was addressed to evaluate the temporal and spatial changes in regional cerebral blood flow (rCBF) of patients with neuropsychiatric systemic lupus erythematosus (NPSLE). Our objective was to correlate the subtracted SPECT coregistered to MRI features (SISCOM) with demographic, clinical and laboratory findings to shed light upon the pathophysiological evolution of the NPSLE. Twenty-six NPSLE patients with MRI and pre- and post-treatment brain SPECT with [99mTc]Tc-ECD. SISCOM features were categorized as improvement, worsening, activation and/or deactivation of rCBF findings. Patients mean age of 43.19 years and 65.38% white were evaluated. The patients mean age at onset of SLE was 26.05 and 42.29 for NPSLE. The mean time between the onset of SLE and first NPSLE symptoms was 05.57 years. The disease has already been initiated as NPSLE in 4 patients. The SLEDAI average score was 31.69 and the SLICC/ACR-DI score was 06.96. The patients underwent an average of 09.23 cyclophosphamide. The SISCOM findings showed functional and pathological states on different brain regions. The rCBF changes were not associated with index scores. There was, however, a trend towards an association between lower SLEDAI scores with improvement and higher SLEDAI with worsening in SISCOM, Also a trend of association between lower SLICC score with improvement, and higher SLICC with worsening. The female gender was predictive of activation and worsening, separately, and deactivation and worsening in a set. Non-white patients were predictive of worsening. The seizure was predictive of deactivation separately, and deactivation and worsening in a set. Finally, normal C3 was a predictor of improvement. The present study showed dynamic brain changes in NPSLE patients. SISCOM technique showed improved rCBF in some brain areas, and worsening, activation and deactivation in others. There were associations between rCBF changes and gender, skin colour and complement C3 and association trends with SLEDAI and SLICC scores.

## Introduction

The systemic lupus erythematosus (SLE) is an inflammatory autoimmune disease that targets many organs and systems, including the nervous system. Neuropsychiatric systemic lupus erythematosus (NPSLE) can affect 12.00% to 75.00% of SLE patients and involve both central and peripheral nervous systems. Cognitive dysfunctions are the most common manifestations, followed by psychosis or mood disorder, cerebrovascular disease, convulsions and headaches (Jafri et al. 2017; Govoni et al. 2016). Although neuropsychiatric (NP) manifestations are frequent, they still constitute a challenge for behavioural and neuroimaging studies (Netto et al. 2013). NP manifestations can precede the onset of SLE or occur at any time during its course (Vandam et al. 1994), and can be single or multiple neurological events in the same individual (Liang et al. 1999). The two most important processes under the etiological aspect of NPSLE are changes in rCBF due to the occlusion of vessels that irrigate the nervous tissue, or interactions of autoantibodies with neurons and glia (Hirohata 2018). Despite the efforts to determine the diagnosis of NPSLE, there is still no standard gold method for the diagnosis and management of the symptoms presented (Appenzeller et al. 2007; Castellino et al. 2011).

The ACR committee responsible for the classification of syndromes in NPSLE considers that structural (Magnetic Resonance Imaging, MRI) and functional brain imaging (Single Photon Emission Computed Tomography, SPECT) may become the gold standard method for the classification of NPSLE and are essential components in some case definitions (Liang et al. 1999). The MRI is widely used to evaluate NPSLE patients, presenting variable sensitivity, being abnormal in 15.0 to 78.0% of patients, and having low specificity, being abnormal in 25.0 to 50.0% of patients with SLE, without NP manifestation (Zardi et al. 2014). It is quite useful in cases of focal impairment of NPSLE (Tan et al. 2018) and less effective in cases of diffuse involvement (Levy and Carvalho 2017). Another significant limitation is the difficult differentiation between new or active from old or sequel injuries.

The brain SPECT evaluates the regional cerebral blood flow (rCBF) and contributes to diagnosing cerebrovascular diseases, dementia, epilepsy, movement disorders and psychiatric, vascular and degenerative diseases (Matsumoto et al. 2017). It has been known that immune dysfunction in active SLE affects the brain by different mechanisms, either the presence of circulating autoantibodies, entry of pro-inflammatory cytokines and chemokines into the cerebrospinal fluid or even the rupture of the blood–brain barrier (Duarte-Delgado et al. 2019). Brain SPECT has shown abnormalities in the central nervous system (CNS) in the SLE (Appenzeller et al. 2007), with a higher correlation with clinical findings in NPSLE, than MRI. The most common SPECT findings in NPSLE patients are focal, diffuse or multifocal areas of decreased rCBF, related to the severity and activity of NPSLE. Lesions are commonly seen in middle cerebral artery territory, followed by parietal, frontal and temporal lobes, and basal ganglia (Long et al. 2018). In the context of the pathophysiological investigation, the subtraction ictal SPECT coregistered to MRI (SISCOM) can be used beyond epilepsy and may contribute to the temporal evaluation of brain injuries by NPSLE (Aupy et al. 2018). Only case reports have shown the role of SISCOM in NPSLE (Trevisan et al. 2019). The presence of SPECT changes with normal MRI may be predictive of CNS involvement even in the absence of current NP manifestations (Zardi et al. 2014).

This study was addressed to evaluate the temporal and spatial changes in regional cerebral blood flow of patients with NPSLE. Our objective was to correlate SISCOM features with demographic, clinical and laboratory findings to shed light upon the pathophysiological evolution of the NPSLE.

## Patients and methods

### Patients

Twenty-six patients with NPSLE were evaluated by the rheumatology division and the nuclear medicine and PET/CT laboratory of our university hospital. All patients were diagnosed by the criteria of the SLICC/ACR-DI (Systemic Lupus International Collaborating Clinics/American College of Rheumatology—Damage Index), and the disease activity was assessed by the SLEDAI index (Systemic Lupus Erythematosus Disease Activity Index). All patients underwent brain SPECT with [^99m^Tc]Tc-ECD pre- and post-cyclophosphamide and methylprednisolone pulse therapy and in time close to the brain MRI.

### Clinical, MRI and laboratory exams

All patients underwent clinical examinations, determination of anti-DNA antibodies and antinuclear factor antibody (FAN), and the majority of patients to the levels of serum complement C3 and C4, anti-P-ribosomal (P0, P1, P2), anti-Sm and anti-Sm/RNP antibodies, anti-SS-A (RO) and anti-SS-B (LA) antibodies, IgG and IgM anticardiolipin antibodies, b2-glycoprotein I (b2GPI)-dependent, anti-Chromatin antibody, anti-Nucleosome IgG antibody, anti-TPO antibody anti-Peroxidase and anti-β2-glycoprotein-I (B2-GPI).

MRI was acquired using the 3.0-T MRI system Philips Achieva (Philips Medical Systems, Best, The Netherlands), between 2010 and 2019, according to protocols specifically designed for brain studies: Dual echo FSE (fast spin echo) with 1 mm slice thickness, 3D T1-weighted, high-resolution 3D T1-weighted 3D MPRAGE, 3D T2-weighted images, 3D fast T2-weighted FLAIR with fat suppression, axial T2-weighted TSE, DWI (Diffusion Weighted Imaging), axial SWI (Susceptibility Weighted Magnetic Resonance Imaging), non-contrast-enhanced 3D TOF MRA (Magnetic Resonance Angiography).

### SPECT protocol

All patients underwent brain SPECT after the intravenous injection of 1,110 MBq (30 mCi) of the tracer Technetium-99 m Ethyl Cysteinate Diethyl Ester ([^99m^Tc]Tc-ECD). Injection was performed when patients were at rest, with eyes open, in a quiet and darkroom, refrained from moving, talking and listening. SPECT scans were acquired in a double-headed SPECT/CT Philips Brightview XCT (Philips Healthcare, Cleveland, Ohio, USA), with a low energy high-resolution collimator (LEHR), photopeak centred on 140 keV and acceptance window of 20.00%, 64 projections per head over 360°, on a 128 × 128 matrix, acquisition time of 30 min and about 100,000 counts/projection/head. Projections were reconstructed using the ordered subset expectation maximization (OSEM), applying Butterworth filter (order 2, cut-off frequency 0.28), and photon attenuation correction by uniform method (Chang, pixel size 2.13, coefficient 0.12/cm). Images were then reconstructed in transaxial slices parallel to the orbito-meatal line, from which coronal and sagittal sections were produced.

### SISCOM analysis

The SISCOM was performed using the ANALYZE © 10.0 Software (AnalyzeDirect, Inc., Overland Park, Kansas, USA), following the methodology adapted from previous studies (Wichert-Ana et al. 2008). The first and second SPECTs, whether pre and post-treatment, or performed between different clinical pictures, were registered using an alignment algorithm based on mutual information. The first SPECT was subtracted from the second one, and the signal difference was transformed into z-score maps, using the mean and standard deviation of the differences in all brain voxels, resulting in images of increase or decrease of rCBF. This quantification matrix was fused with the patient's MRI, in order to provide functional and anatomical information in the same image. After functional overlap, only images with significant rCBF changes, i.e. more than 2 standard deviations above or below the mean, were exhibited.

SISCOM findings were classified into four groups. *Improvement* was the group of patients who presented increased rCBF in the second SPECT on brain regions that showed decreased rCBF in the first SPECT. Clinically, it means a reperfusion of an ischemic or hypofunctioning brain region after treatment. *Worsening* was the group with decreased rCBF in the second SPECT on a brain region with normal rCBF in the first SPECT. Clinically, it means an ischemic insult or hypofunctioning status of a brain region after treatment. *Activation* was the group with increased rCBF in the second SPECT on a brain region that presented normal rCBF in the first SPECT. Clinically, it means a previously normal brain region that evolved to hyperemia or hyperfunctioning status after treatment. ***Deactivation*** was the group with increased rCBF in the first SPECT on a brain region that presented normal rCBF in the second SPECT. Clinically, it means a previously hyperemic or hyperfunctioning brain region that evolved to normal status after treatment.

### Statistical analysis

Statistical analysis was performed using IBM SPSS Statistics for Windows, Version 23 (IBM SPSS Statistics for Windows, Launched in 2015, IBM Corporation, Armonk, NY, USA). Binary logistic regression analysed if clinical, laboratory and complement (C3 and C4) variables were predictive of SISCOM findings. Only the variables that showed significant differences between patients with normal and abnormal evolution (*p* < 0.05) were evaluated. The Mann–Whitney test (Wilcoxon rank-sum test) analysed whether there were significant differences between nonparametric variables (*p* < 0.05).

## Results

### Demographic data

Table 1 summarizes demographic and clinical features of the NPSLE patients. Twenty-six patients (19 females, 73.07%; 7 males, 26.93%), with mean age of 43.19 years (SD 11.33; 95% CI 38.61–47.77), ranging from 20 to 70 years, were evaluated. Sixteen women had normal pregnancy (69.56%), one had abortion (04.36%), and six were nulliparous (26.08%). Most patients were self-declared white (*n* = 17; 65.38%), followed by mixed race, brown colour, and black (non-white; *n* = 9; 34.62%). The patient's mean age at onset of SLE was 26.05 (SD 11.04) years, ranging from 8 to 51 years, and for NPSLE was 42.29 (SD 13.49) years, ranging from 11 to 59 years. The mean time between the onset of SLE and first NPSLE symptoms was 05.57 (SD 5.02) years, ranging from zero to 22 years. The disease has already been initiated as NPSLE in 04 patients (15.38%).

**Table 1:** Demographic, clinical and laboratory comparisons among patients with SISCOM

Patients characteristics	Total (*n* = 26)	Amelioration (*n* = 15, 57.69%)	Activation (*n* = 12, 46.15%)	Deactivation (*n* = 8, 30.77%)	Worsening (*n* = 6, 23.07%)	Binary logistic regression	Binary logistic regression		Mann-Whiney Test
							Amelioration and activation	Deactivation and worsening	
Sex, female/male, *n* (%)	19 (73.07)/7 (26.93)	10 (66.67)/05 (33.33)	08 (66.70)/04 (33.33)*	05 (62.50)/03 (37.50)	04 (66.67)/02 (33.33)**	* [*X*^2^(1) = 5.804; *p* = 0.041, *R*^2^ Negelkerke = 0.267] ** [*X*^2^(1) = 9.781; *p* = 0.008, *R*^2^ Negelkerke = 0.475]	NS	[*X*^2^(1) = 5.804; *p* = 0.041, *R*^2^ Negelkerke = 0.282]	–
Age (years), mean (SD)	43.19	44.13	40.91	43.48	43.03	NS	NS	NS	–
Race/ethnicity *n* (%) (Caucasians/non-Caucasians)	17 (65.38)/09 (34.62)	09 (60.00)/06 (40.00)	08 (66.67)/04 (33.33)	04 (50.00)/04 (50.00)	04 (66.67)/02 (33.33)*	*[*X*^2^(1) = 8.119; *p* = 0.015, R2 Negelkerke = 0.406]c	NS	NS	–
Age at SLE onset (mean, years)	26.05	28.13	26.33	27.00	20.67	–	–	–	NS
Age at NPSLE onset (mean, years)	42.29	46.83	42.08	43.25	47.83	–	–	–	NS
Interval between SLE and NPSLE first symptoms (mean, years)	05.57	04.80	07.25	07.12	02.67	–	–	–	NS
SLEDAI, mean (SD)	31.69	33.80	29.83	29.00	35.00	–	–	–	NS
SLICC/ACR-DI mean (SD)	06.96	06.73	06.67	06.25	07.83	–	–	–	NS
Number of Pulse Therapies, mean (SD)	09.23	08.80	09.92	09.50	08.33	–	–	–	NS
Normal MRI *n* (%)	06 (23.07%)/20 (76.93)	01 (06.67)	05 (41.67)	04 (50.00)	00 (00.00)	–	–	–	–
Abnormal MRI *n* (%)	06 (23.07%)/20 (76.93)	14 (93.33)^#^	07 (58.33)	04 (50.00)^#^	06 (100.00)	–	–	–	–
NP Manifestation n (%)***									
Psychosis	17 (65.38)	08 (53.33)	10 (83.33)	06 (75.00)	03 (50.00)	NS	NS	NS	–
Bipolar disorder	01 (3.84)	02 (13.33)	00 (00.00)	01 (12.50)	01 (16.67)	NS	NS	NS	–
Depression	07 (26.92)	03 (20.00)	05 (41.67)	03 (37.50)	01 (16.67)	NS	NS	NS	–
Vasculitis	02 (7.69)	02 (13.33)	00 (00.00)	00 (00.00)	02 (33.33)	NS	NS	NS	–
Seizure	08 (30.77)	04 (26.67)	03 (25.55)	02 (25.00)	02 (33.33)	NS	NS	NS	–
Anxiety	06 (23.07)	03 (20.00)	03 (25.00)	02 (25.00)	02 (33.33)	NS	NS	NS	–
Headache	02 (7.69)	01 (06.67)	01 (08.33)	01 (12.50)	01 (16.67)	NS	NS	NS	–
Epilepsy	01 (3.84)	01 (06.67)	00 (00.00)	00 (00.00)	01 (16.67)	NS	NS	NS	–
Stroke	01 (3.84)	01 (06.67)	00 (00.00)	00 (00.00)	01 (16.67)	NS	NS	NS	–
*Autoantibodies—n (%)*									
Native anti-DNA reagent/not reagent	13/13 (73.07/26.93)	06 (40.00)/09 (60.00)	08 (66.67)/04 (33.33)	04 (50.00)/04 (50.00)	02 (33.33)/04 (66.67)	NS	NS	NS	–
ANA reagent/not reagent	19/07 (73.07/26.93)	10 (66.67)/05 (33.33)	10 (83.33)/02 (16.67)	06 (75.00)/02 (25.00)	03 (50.00)/03 (50.00)	NS	NS	NS	–
ACA IgG positive/negative (RV > 10 GPL/ml)	09/13 (30.76/69.24)	06 (37.50)/07 (46.67)	04 (3.33)/06 (50.00)	03 (37.50)/03 (37.50)	02(33.33)/03(50.00)	NS	NS	NS	–
ACA IgG not done	04 (15.38)	02 (13.33)	02 (16.67)	02 (25.00)	01 (16.67)	–	–	–	–
ACA IgM positive/negative (RV > 7 MPL/ml)	12/14 (46.15/53.85)	09 (60.00)/04 (26.67)	06 (50.00)/03 (25.00)	02 (25.00)/04 (50.00)	02(33.33)/03(50.00)	NS	NS	NS	–
ACA IgM not done	04 (15.38)	02 (13.33)	03 (25.00)	02 (25.00)	01 (16.67)	–	–	–	–
P2GPI IgG positive/negative (RV > 5 GPL/ml)	05/11 (31.25/68.75)	04 (26.67)/06 (40.00)	02 (16.67)/08 (66.67)	02(25.00)/03(37.50)	01(16.67)/04(66.67)	NS	NS	NS	–
P2GPI IgG not done	10 (38.46)	05 (33.33)	02 (16.67)	03 (37.50)	01(16.67)	–	–	–	–
P2GPI IgM positive/negative (RV > 5 MPL/ml)	07/09 (43.75/56.25)	06 (40.00)/04 (26.67)	04 (33.33)/06(50.00)	01 (12.50)/03(37.50)	01 / 04(66.67)	NS	NS	NS	–
P2GPI IgM not done	10 (38.46)	05 (33.33)	02 (16.67)	04 (50.00)	01(16.67)	–	–	–	–
SS-B (LA) positive/negative (RV > 20 U/ml)	01/06 (14.29/85.71)	01 (06.67)/06 (40.00)	00 (00.00)/05 (41.67)	00 (00.00)/05 (50.00)	01 (16.67)/01 (16.67)	NS	NS	NS	–
SS-B (LA) not done	19 (73.08)	08 (53.33)	07 (58.33)	07 (87.50)	04 (66.67)	–	–	–	–
SS-A (RO) positive/negative (RV > 80 U/ml)	03/07 (30.00/70.00)	02 (13.33)/08 (53.33)	00 (00.00)/06 (50.00)	01 (12.50)/04 (50.00)	00 (00.00)/02 (33.33)	NS	NS	NS	–
SS-A (RO) not done	16 (61.53)	05 (33.33)	06 (50.00)	03 (37.50)	04 (66.67)	–	–	–	–
*Complement—n(%)*									
C3 normal (RV 0.9–1.4 U/ml)	16 (61.50)	09 (60.00)*	07 (58.33)	02 (25.00)	06 (100.00)	*[*X*^2^(1) = 6.168; *p* = 0.021, *R*^2^ Negelkerke = 0.303]	NS	NS	–
C3 altered / not done	08 (30.76)/02 (07.69)	03/02 (13.33)	05 (41.67)/00 (00.00)	06 (75.00)/00 (00.00)	00 (00.00)/00 (00.00)	–	–	–	–
C4 normal (RV 0.1–0.4 U/ml)	16 (61.50)	10 (66.67)	07 (58.33)	06 (75.00)	04 (66.67)	NS	NS	NS	–
C4 altered / not done	08 (30.76)/02 (07.69)	04 (26.67)/01 (06.67)	05 (41.67)/00 (00.00)	02 (25.00)/00 (00.00)	02 (33.33)	–	–	–	–

*SD* standard deviation, *NS* not significant, *SLE* systemic lupus erythematosus, *NPSLE* neuropsychiatric systemic lupus erythematosus, *SLEDAI* systemic lupus erythematosus disease activity index, *SLICC/ACR-DI *Systemic Lupus International Collaborating Clinics/American College of Rheumatology

^#^Chi-square test *p* = 0,05

***It is noteworthy that the same patient may have overlapping SISCOM findings

### Clinical and laboratory data

The average score on SLEDAI was 31.69 (SD 10.33; 95% CI 27.51–35.86), ranging from 11 to 51 scores, and the SLICC/ACR-DI score was 06.96 (SD 2.37; 95% CI 6.00–7.92), ranging from 2 to 11 scores. The patients underwent an average of 09.23 pulse therapy sessions (SD 3.12; 95% CI 7.96–10.49), ranging from 4 to 15 sessions.

Seventeen (65.38%) patients presented psychosis, 01 (03.84%) bipolar disorder, 07 (26.92%) depression, 02 (07.69) vasculitis, 08 (30.77) seizure, 06 (23.07) anxiety, 02 (07.69) headache, 01 (03.84) epilepsy and 01 (03.84) stroke.

All patients underwent native anti-DNA and ANA laboratory tests. For Native anti-DNA and ANA, 13 (50%) and 19 (73.07%) patients were reactive, respectively. For ACA IgG and IgM, nine (30.76%) and 13 (50.09%) patients were positive, respectively. For anti-β2GPI IgG and IgM, five (31.25%) and seven (43.75%) patients were positive, respectively. For anti-SS-B (LA) and anti-SS-A (RO), one (14.29%) and three (30.00%) patients were also positive, respectively. Complement levels were altered in 11 (42.31%) patients for C3, and in 8 (30.77%) for C4.

### SISCOM findings

The MRI was normal in 6 (23.08%) patients and abnormal or with nonspecific findings in another 20 (76.92%). SISCOM agreed with MRI findings in 13 patients (50.00%). None of the NP manifestations correlated with the SISCOM findings, i.e. the evolution of rCBF changes (SISCOM) was not influenced by the type of NP manifestation.

Regarding the SISCOM findings, 15 (57.69%) patients presented improvement, 12 (46.15%) activation, 8 (30.77%) deactivation and 6 (23.07%) presented worsening. These categories were not associated with the degree of activity [SLEDAI, Mann–Whitney test; improvement, *U* = 60.5000, *p* = 0.252; activation, *U* = 66,000, *p* = 0.353; deactivation, *U* = 51,500, *p* = 0.254 and worsening, *U* = 51,000, *p* = 0.583] or disease-related damage [SLICC, Mann Whitney Test; improvement, *U* = 65,500, *p* = 0.371; activation, *U* = 67,000, *p* = 0.375; deactivation, *U* = 47,500, *p* = 0.167 and worsening, *U* = 56,000, *p* = 0.805]. However, there was a trend towards an association between lower disease activity (SLEDAI 33.80) and improvement, and greater activity (SLEDAI 35.00) with worsening [Mann Whitney test, *U* = 56,000; *p* = 0.072]. There was also a trend of association between lower damage associated with LESNP (SLICC 6.73) with improvement, and greater damage (SLICC 7.83) with worsening rCBF (Mann–Whitney test, *U* = 56,000, *p* = 0.085).

Binary logistic regression showed that the model containing female gender was significant for activation [*X*^2^(1) = 5.804; *p* = 0.041, *R*^2^ Negelkerke = 0.267] and worsening [*X*^2^ (1) = 9.781; *p* = 0.008, *R*^2^ Negelkerke = 0.475] in SISCOM, but not significant for amelioration and deactivation. The female gender was predictive of activation (OR = 0.091; IC 95% = 0.009—0.906) and worsening (OR = 28.333; IC 95% = 2.389–336.008), but not predictive of amelioration or deactivation. The model containing female gender was also significant for the finding set of deactivation and worsening [*X*^2^(1) = 5.804; *p* = 0.041, *R*^2^ Negelkerke = 0.282], but not significant for the set of activation and worsening. The female gender was predictive of the deactivation and worsening set (OR = 11,000; IC 95% = 1.103–109.674), and not predictive of the activation and worsening set.

Binary logistic regression showed that the model containing non-white races was significant for worsening in the SISCOM [*X*^2^(1) = 7.279; *p* = 0.020, *R*^2^ Negelkerke = 0.387], but not for amelioration, activation or deactivation. Non-white races were predictive of worsening in SISCOM (OR = 17,500; IC 95% = 1.560–196.319), but not predictive of improvement, activation or deactivation.

Binary logistic regression of the 24 patients who measured the presence of complement C3 showed that the model containing the normal result (RV 0.9–1.4 U / ml) was significant for improvement in SISCOM [*X*^2^(1) = 7.279; *p* = 0.021, *R*^2^ Negelkerke = 0.303], but not for the worsening, activation or deactivation. Normal C3 was a significant predictor of improvement in SISCOM (OR = 8.889; IC 95% = 1.397—56.575), but not for worsening, activation or deactivation. Normal C4 was not a significant predictor of any SISCOM finding.

**Fig. 1:**
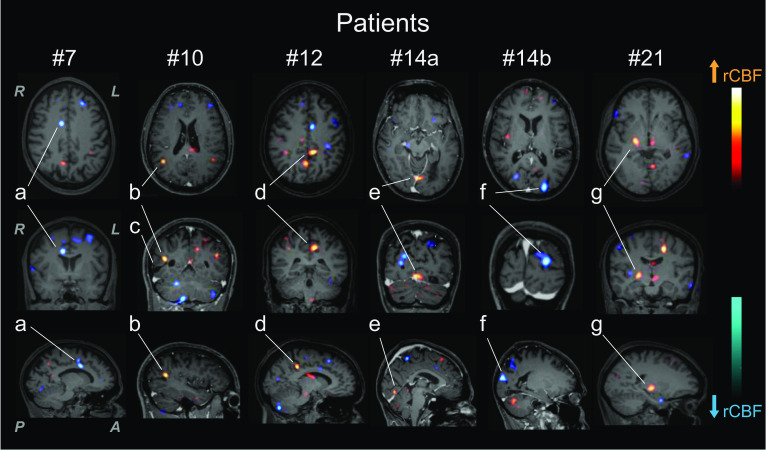
Subtraction SPECT coregistered to MRI (SISCOM). *Patient #7* presented decreased rCBF in the anterior cingulate cortex (a), associated with recovered anxiety, addiction and psychosis after pulse therapy. *Patient #10* presented increased rCBF in the right inferior parietal lobe (b) adjacent to a vasculitic insult (c), possibly due to luxury perfusion of the neighbouring cortex after treatment, and associated with the recovery of memory and hallucinations. *Patient #12* presented increased rCBF in the precuneus, posterior cingulate cortex (d), and hypothalamus, associated with post-treatment persistent psychosis, aggressivity, and behaviour modification. *Patient #14* presented two functional status after a left posterior cortex stroke: increased rCBF (e) (#14a) in the left medial occipital lobe (reperfusion) possibly related to post-treatment recovery from the hallucinations, and decreased rCBF (f) (#14b) in the posterior occipital lobe (worsening of ischemia). *Patient #21* presented increased rCBF overlapping with the infarcted right basal ganglia (g), possibly representing luxury perfusion, and associated with the improvement in depressive symptoms

Binary logistic regression of the NP manifestations showed a trend between seizure and deactivation and worsening group [*X*^2^(1) = 3.798; *p* = 0.062, *R*^2^ Negelkerke = 0.185; OR = 5.133; IC 95% = 0.922–28.570] and deactivation group [*X*^2^(1) = 3.665; *p* = 0.096, *R*^2^ Negelkerke = 0.185; OR = 7.000; IC 95% = 0.709–69.121], but not for the amelioration, activation and worsening (Fig. 1).

## Discussion

The present study evaluated the temporal and spatial changes of rCBF through subtracted serial brain SPECT in patients with NPSLE. The SISCOM findings showed functional (activation, deactivation) and pathological (improvement, worsening) states on different brain regions. The rCBF changes were not associated with SLEDAI or SLICC scores. There was, however, a trend towards an association between lower SLEDAI score with improvement, and higher SLEDAI with worsening in SISCOM; also, a trend of association between lower SLICC score with improvement, and higher SLICC with worsening in SISCOM. The female gender was predictive of activation and worsening, separately, and deactivation and worsening in a set. Non-white races were predictive of worsening in SISCOM. Finally, normal C3 was a predictor of improvement in SISCOM after treatment.

The SISCOM showed that 57.69% of patients presented improvement, 46.15% activation, 30.77% deactivation and 23.07% presented worsening of rCBF. These findings agreed with MRI in only 50% of patients. None of the neuropsychiatric manifestations correlated with the SISCOM findings. A correlation between focal lesions on MRI and areas of decreased rCBF in SPECT have been reported in 38.70% of patients with LESNP, and a lack of correlation in 61.29% (Oku et al. 2003). A previous study showed decreased rCBF in 90% of patients with NPSLE and in 20.00% of patients with SLE only (Huang et al. 2002). Most SPECT changes have been observed before treatment, notably in the parietal lobes (91.70%) and less in the cerebellum (25.00%), with complete recovery of rCBF after therapy in 83.30% of NPSLE patients (Liu et al. 2003). Nine of our 26 patients presented decreased rCBF in parietal lobes that evolved to normal rCBF in 8 (88.88%) after treatment. In NPSLE patients, bilateral decreased rCBF was found in frontal lobes in up to 81.10% of patients with cognitive impairment (Driver et al. 2008), and decreased rCBF in the precuneus in those with memory impairment (Oh et al. 2011). Previously, we reported an aphasic female NPSLE patient who evolved with reperfusion in the Broca's area after a vasculitic insult, documented by SISCOM (Trevisan et al. 2019).

The effect of gender on NPSLE may be related to genetic and environmental factors (Ginzler and Dooley 2014; Isenberg 2012), and female sex hormones (oestrogen), on the immune system (O’Neill and Cervera 2010). The female predominance is 9:1 (Falasinnu et al. 2018; Nusbaum et al. 2020), but in our study it was 3:1. Our female sample showed more functional activation in the second SPECT, and consequently in SISCOM, but with decreased rCBF compared to the male sample. This finding contrasts previous studies where men with SLE form an unusual group of inflammatory disease, more aggressive, with acute psychosis and seizure, and more severe sequelae than women (Falasinnu et al. 2018; Lisnevskaia et al. 2014; Rees et al. 2017).

The incidence and prevalence of SLE are higher in non-Caucasian (African, African-American and Hispanic) than Caucasian (white) races (O’Neill and Cervera 2010; Lisnevskaia et al. 2014). Our non-white patients showed worsening rCBF in SISCOM compared to white. Genetic studies point to genetically determined ancestry and environmental factors as responsible for these ethnicity-related biological processes (Lewis and Jawad 2017). There has been a considerable increase in SLE rates in non-white patients recently, which may increase diagnostic and therapeutic challenges (Phuti et al. 2019).

Complement C3 is a complex system of proteins associated with cell membranes, contributing to the clearance of immune complexes and inflammatory processes. C3 deficiency is associated with severe inflammatory tissue destruction in NPSLE (Dossantos and Wiethölter 2021; Utiyama et al. 2004). This deficiency compromises the activities related to opsonization and phagocytosis, causing greater susceptibility to infections and being related to the worsening of NPSLE. Thus, normal C3 dosage favours the improvement in the inflammation process in these patients (Utiyama et al. 2004). In our study, normal C3 was interestingly associated with improved rCBF in SISCOM, confirming the role of C3 in the resolution of neuroinflammation in NPSLE.

### Limitations of the study

In our study, the lack of correlation between SISCOM findings and neuropsychiatric manifestations, and only trends of association between the SLEDAI and SLICC scores with SISCOM may be due to the small sample of patients. It was not possible to compare our NPSLE patients with those with only SLE. Our study was retrospective and the SPECT was successfully used for more than a decade to evaluate NPSLE patients only.

## Conclusions

The present study showed dynamic brain changes in cerebral blood flow in NPSLE patients. SISCOM technique showed improved rCBF in some brain areas, and worsening, activation and deactivation in others. There were associations between rCBF changes and gender, races and complement C3, and association trends with SLEDAI and SLICC scores. Future studies should correlate these rCBFs with neuropsychiatric symptoms in larger samples and perform clinical trials that evaluate the efficacy of therapies in light of rCBF changes.

## Data Availability

All data generated or analysed during this study are included in this published article [and its supplementary information files].
